# Higher Sex‐Reversal Rate of Urban Frogs in a Common‐Garden Experiment Suggests Adaptive Microevolution

**DOI:** 10.1111/eva.70093

**Published:** 2025-04-07

**Authors:** Veronika Bókony, Emese Balogh, Zsanett Mikó, Andrea Kásler, Zoltán Örkényi, Nikolett Ujhegyi

**Affiliations:** ^1^ Department of Evolutionary Ecology Plant Protection Institute, HUN‐REN Centre for Agricultural Research Budapest Hungary; ^2^ Molecular Ecology Research Group, Department of Zoology University of Veterinary Medicine Budapest Budapest Hungary; ^3^ Department of Systematic Zoology and Ecology ELTE Eötvös Loránd University Budapest Hungary; ^4^ Doctoral School of Biology, Institute of Biology ELTE Eötvös Loránd University Budapest Hungary; ^5^ Department of Wildlife Biology and Management, Institute for Wildlife Management and Nature Conservation Hungarian University of Agriculture and Life Sciences Gödöllő Hungary

**Keywords:** adaptation, environmental change, microevolution, phenotypic plasticity, temperature‐dependent sex determination, urbanization

## Abstract

Ectothermic vertebrates with genotypic sex determination may adjust their sexual phenotype to early‐life environmental conditions by sex reversal, and theoretical models predict diverse consequences for population dynamics and microevolution under environmental change. Environments that frequently expose individuals to sex‐reversing effects may select for or against the propensity to undergo sex reversal, depending on the relative fitness of sex‐reversed individuals. Yet, empirical data on the adaptive value and evolutionary potential of sex reversal is scarce. Here we conducted a common‐garden experiment with agile frogs ( *Rana dalmatina* ) that respond to larval heat stress by sex reversal, to test whether sex‐reversal propensity has changed via microevolution in populations that live in anthropogenic habitats where potentially sex‐reversing heat events are more frequent, compared to populations that live in cooler woodland habitats. Furthermore, to infer the adaptive value of sex reversal, we compared fitness‐related traits between heat‐exposed genotypic females that phenotypically developed into males (sex‐reversed) or females (sex‐concordant). We found that the frequency of sex reversal varied between sibgroups and was higher in the sibgroups originating from anthropogenic habitats, regardless of the thermal environment they had been exposed to during the larval sex‐determination period. Among heat‐exposed animals, time to metamorphosis was similar between sex‐reversed individuals and sex‐concordant females, but the former reached larger body mass by the end of the experiment than the latter, approaching the mass of sex‐concordant males. These results suggest that sex‐reversal propensity may have increased in anthropogenic environments by adaptive microevolution, potentially to minimize the fitness cost of reduced growth caused by heat events. Thus, environmental sex reversal has the potential to provide an adaptive strategy for ectothermic vertebrates to cope with challenges of the Anthropocene. Such knowledge on the causes and consequences of sex reversal will help pinpoint which populations are most threatened by extinction due to climatically influenced sex determination.

## 1 Introduction

**FIGURE 1:**
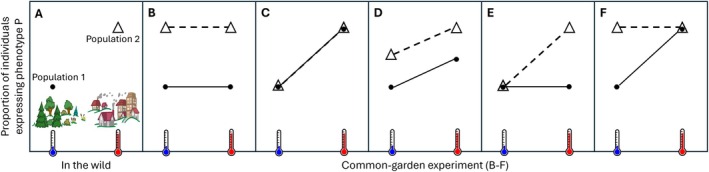
Schematic illustration of mechanisms of phenotypic divergence between populations living in different environments. In this hypothetical example (A), Population 1 lives in a cool woodland habitat while Population 2 lives in a warm urban habitat, and the proportion of individuals expressing phenotype P is higher in the latter than in the former. When raised in a common‐garden experiment (B–F), the phenotypes of individuals originating from different populations depend on whether the higher propensity to express phenotype P in the urban population is due to microevolution (B), phenotypic plasticity (C), the combination of the latter two (D), or evolved plasticity in the urban population (E) or evolved loss of plasticity in the urban population (F).

Ongoing anthropogenic environmental changes are exposing life on Earth to systematic alterations in biotic and abiotic factors, and this has been accompanied by a multitude of phenotypic changes in wild populations (Alberti et al. 2017; Johnson and Munshi‐South 2017; Parmesan 2006; Seress and Liker 2015; Turcotte et al. 2017). Although some of these phenotypic changes may be neutral to fitness or even maladaptive, like the phenological mismatches between trophic levels (Renner and Zohner 2018), a growing body of evidence demonstrates their adaptive value (Lambert et al. 2021; Liker 2020). The mechanisms underlying these phenotypic changes are of particular importance because different processes may enable the most efficient phenotypic adaptation under different circumstances, such as different generation times and gene‐flow levels (Reed et al. 2011). Microevolution can result in local adaptation (Figure 1A,B); for example, fish populations in polluted habitats have evolved tolerance against deadly contaminants (Whitehead et al. 2012). This requires heritable variation in the phenotypic trait, although transgenerational changes may also occur by epigenetic responses to the environment (Hammond et al. 2016). Phenotypic plasticity during individuals' lifetimes can also produce adaptive phenotypes (Figure 1C); for example, urban animals often show reduced behavioral fear responses to repeated human disturbance, and habituation can play an important role in this (Samia et al. 2015). Microevolution and phenotypic plasticity can also act together (Figure 1D); for example, both processes seem to have contributed to the higher heat tolerance of water fleas in warm urban ponds (Brans, Stoks, et al. 2018). Furthermore, plasticity itself can evolve (Figure 1E,F), resulting in different reaction norms in different populations (Crispo et al. 2010; Matesanz and Ramírez‐Valiente 2019). These processes determine whether populations can adapt fast enough to contemporary rapid environmental changes, which is one of the most pressing questions for the conservation of biodiversity and ecosystem services.

An interesting form of phenotypic plasticity is environmentally sensitive sex determination. Its most well‐known form is temperature‐dependent sex determination (TSD), such as in crocodilians and many turtles and fish, whereby the individual's phenotypic sex is determined by the thermal conditions experienced during a sensitive window during early development (Edmands 2021). Both theoretical models and empirical data suggest that TSD is an adaptive strategy when the early environment has sex‐dependent effects on fitness prospects (Bock et al. 2023; Schwanz et al. 2016; Valenzuela 2021). However, under rapid unidirectional environmental change, TSD may become maladaptive by failing to produce one of the two sexual phenotypes altogether (Mitchell and Janzen 2010).

A special kind of environmentally sensitive sex determination is environmental sex reversal, which can occur in species that exhibit genotypic sex determination. In such species, early environmental stimuli may override the effects of sex chromosomes or of a polygenic sex‐determining system, resulting in discordance between phenotypic and genotypic sex (Edmands 2021). Traditionally, it was thought that sex reversal is a rare deviation from the norm or a side effect of unnatural environmental conditions, typically confined to laboratory experiments (Lambert 2015). However, a recent surge of empirical evidence indicates that sex reversal may be much more widespread than previously recognized in ectothermic vertebrates under ecologically relevant circumstances (reviews by Baroiller and D'Cotta 2016; Holleley et al. 2016; Nemesházi and Bókony 2022). Theoretically, sex reversal may be an adaptive sex‐allocation strategy similar to TSD, allowing the individual to express the sexual phenotype that best matches its environment (Geffroy and Douhard 2019). Empirically, however, we know next to nothing about the adaptive value of sex reversal in the wild (Wild et al. 2022), and the scant data from studies done on captive animals are contradictory (Bókony et al. 2021; Holleley et al. 2015; Senior et al. 2012).

Environmental sex reversal is highly relevant for understanding the effects of anthropogenic environmental change because the triggers of sex reversal coincide with the environmental factors that are being most conspicuously altered by humankind, such as thermal and chemical conditions (Baroiller and D'Cotta 2016; Holleley et al. 2016; Nemesházi and Bókony 2022). Theoretical models predict that, under environmental change, sex reversal can have complex and far‐reaching consequences for population persistence and microevolution, and the outcomes depend on many characteristics of the species as well as the environment (Bókony et al. 2017; Grossen et al. 2011; Nemesházi et al. 2021; Schwanz et al. 2020). One such decisive characteristic is the relative fitness of sex‐reversed individuals. If the traditional view is right, and sex reversal is a negative side effect of environmental stress, it might endanger biodiversity by facilitating population collapses in environmentally sensitive species (Nemesházi et al. 2021). However, if sex reversal is adaptive, it might help populations cope with the challenges of ongoing environmental change (Pen et al. 2010; Schwanz et al. 2013).

In both cases, we can expect changes in the propensity for sex reversal in response to anthropogenic environmental change (Nemesházi et al. 2022). If sex reversal is disadvantageous, resistance to it may be adaptive in environments where sex‐reversing stressors are pervasive. For example, if sex reversal is a side effect of heat stress suffered during early ontogeny, individuals that are less prone to heat‐triggered sex reversal may be favored in urban habitats, where temperatures are higher and heat waves are more frequent due to the urban heat island effect (Bókony, Balogh, et al. 2024; Brans, Engelen, et al. 2018). On the other hand, if sex reversal is beneficial under certain conditions, it may be most selected for in environments where such conditions occur often. For example, if early‐life heat stress affects males and females differently, then heat‐induced sex reversal may allow individuals to choose the sexual phenotype that maximizes their fitness given their thermal experiences. This capacity for sex reversal may have the highest pay‐off in environments where heat waves are common, such as urban habitats or agricultural areas where shading vegetation is missing. In contrast, sex‐reversal capacity may be neutral in habitats where heat waves rarely ever happen.

To test these alternative hypotheses, we studied the agile frog ( *Rana dalmatina* ), an emerging model system of ecologically relevant sex reversal. This species is common in Europe, although its numbers are declining; it prefers light woodlands but also occurs in various anthropogenically modified habitats (IUCN SSC Amphibian Specialist Group 2023). It has an XX/XY sex‐chromosome system, and a few‐days heat wave around the middle of larval development causes genotypic females to develop into phenotypic males (Mikó et al. 2021, Ujszegi et al. 2022). In wild populations, 20% of phenotypically male adults are genotypic females, especially in urban and agricultural areas (Nemesházi et al. 2020). Earlier studies that compared fitness‐related traits between sex‐reversed and sex‐concordant agile frogs found heterogeneous results (Bókony et al. 2021; Mikó et al. 2021; Nemesházi et al. 2020), but none of those studies was able to make the most relevant comparison: between heat‐exposed genotypic females that developed the male versus female phenotype (i.e., those that did vs. did not undergo sex reversal in response to the same heat stress). In the present study, we performed a common‐garden experiment by raising agile frogs from eggs collected in anthropogenic habitats and natural woodlands together in captivity, and exposed half of them to a simulated heat wave to measure the rate of sex reversal. We predicted that, if the tendency for sex reversal is selected for in “hot” environments by microevolution, individuals originating from anthropogenic habitats should be more likely to undergo sex reversal than individuals originating from woodlands when experiencing the same thermal conditions during their ontogeny. Furthermore, we measured larval development time and early‐life growth, both of which can affect lifetime fitness in amphibians (Altwegg and Reyer 2003; Smith 1987). We predicted that, if sex reversal is adaptive in agile frogs, the negative effect of heat stress on fitness‐related traits should be smaller in individuals that undergo sex reversal compared to those that do not respond to the heat stress by sex reversal.

## 2 Material and Methods

### 2.1 Experimental Procedures

**FIGURE 2:**
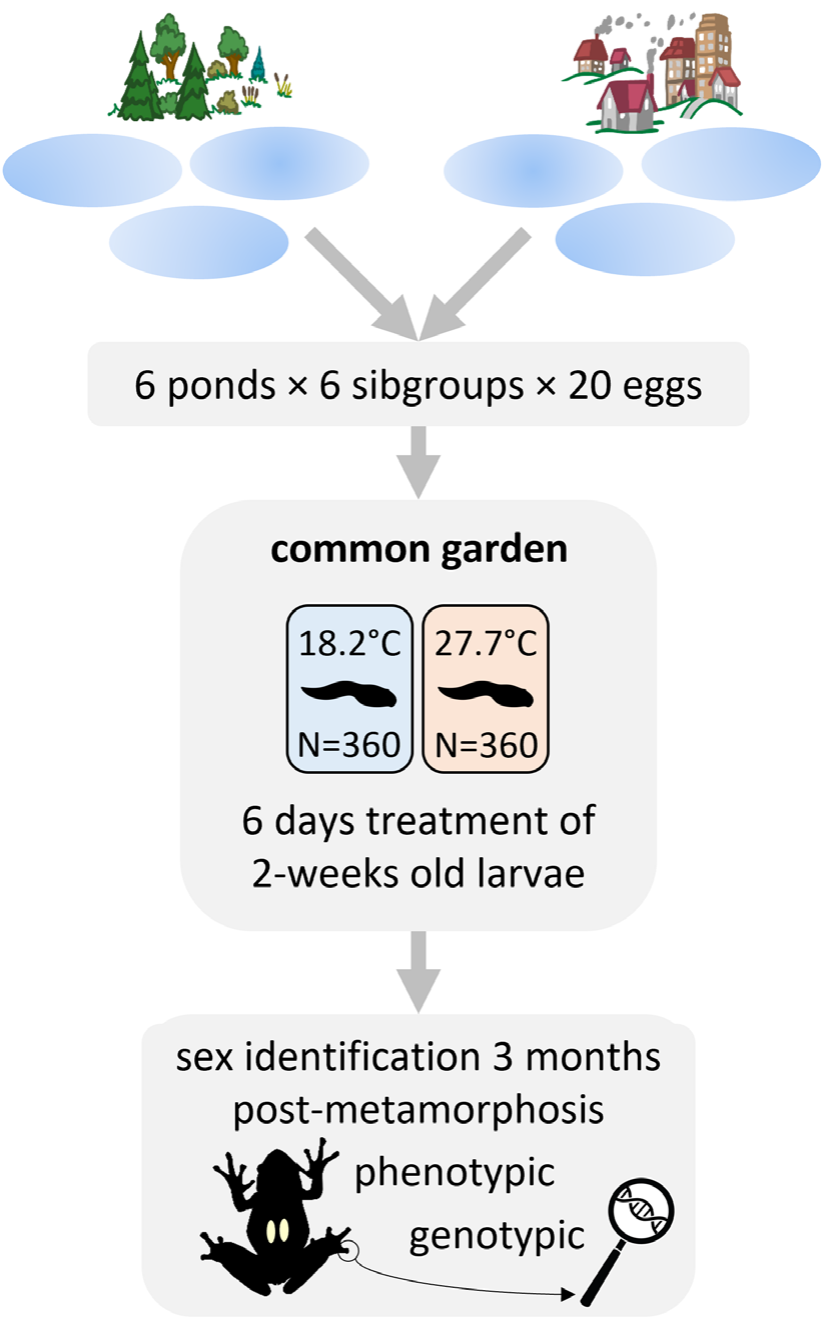
Schematic illustration of our experimental design.

This study was approved by the Ethics Committee of the Plant Protection Institute and licensed by the Environment Protection and Nature Conservation Department of the Pest County Bureau of the Hungarian Government (PE‐06/KTF/00754‐8/2022, PE‐06/KTF/00754‐9/2022, PE‐06/KTF/00754‐10/2022, PE/EA/295‐7/2018, PE/EA/00270‐6/2023). We used six study sites that are described in detail in an earlier study (Bókony, Balogh, et al. 2024): three ponds with < 0.1% anthropogenically modified habitat within 500 m, and three ponds in three different townships with ca. 70% anthropogenically modified land cover (including urban and agricultural areas) within 500 m. Water temperatures in the tadpole developing season are significantly higher in the latter than in the former (Bókony, Balogh, et al. 2024). For simplicity, we are henceforth referring to the three anthropogenic sites as “urban”. We collected freshly spawned eggs from the six ponds (Figure 2) on 13–14 March 2023. From each pond, we took ca. 50 embryos from each of six egg masses and transported them to our laboratory. We kept each group of siblings (henceforth “sibgroup”) in a separate container with ca. 1 cm deep reconstituted soft water (RSW; 48 mg NaHCO_3_, 30 mg CaSO_4_ × 2 H_2_O, 61 mg MgSO_4_ × 7 H_2_O, 2 mg KCl added to 1 L reverse‐osmosis filtered, UV‐sterilized, aerated tap water). Over the course of the study, temperature in the lab was set to gradually increase from 16°C to 22°C (mean ± standard deviation: 19.7°C ± 1.7°C), and we adjusted the photoperiod weekly to mimic the natural dark–light cycles. When the animals reached the free‐swimming state, that is, developmental stage 25 according to Gosner (1960), we started the experiment on 28 March. We haphazardly selected 20 healthy‐looking tadpoles from each sibling group (*N* = 720 in total; Figure 2) and placed them individually in 2‐L plastic rearing containers filled with 1 L RSW, arranged in a randomized block design to ensure that all six populations were homogeneously distributed across the shelves in the laboratory. We changed the rearing water twice a week and fed the tadpoles *ad libitum* with chopped, slightly boiled spinach. The remaining tadpoles were released at their ponds of origin.

Two weeks after starting the experiment, we applied heat treatment for 6 days following previously described methods (Ujszegi et al. 2022). During this period, all tadpoles were kept in 1.7 L RSW, which we changed every other day; at the same times, we fed them a reduced (ca. 1/3) amount of spinach to prevent water fouling and anoxia in the heat treatment. Throughout the treatment period, the tadpole‐rearing containers were kept in groups of 12 in a tray of water that was circulated by a water pump and, for the heat‐treatment group, heated to 27.7°C ± 0.4°C (mean ± SD) by a thermostat aquarium heater. In the control treatment, the water was kept at room temperature, which was 18.2°C ± 0.5°C (mean ± SD) during the treatment period. From each sibling group, we randomly assigned 10 tadpoles to each treatment, resulting in 60 heat‐treated and 60 control tadpoles from each pond (Figure 2).

After the treatment, we raised the tadpoles using the same methods as during their first 2 weeks of larval development. When an individual initiated metamorphosis as marked by the emergence of forelimbs (development stage 42), we measured body mass (±0.1 mg), and we replaced the rearing water with 0.1 L fresh RSW. The containers were slightly lifted on one side to ensure a dry surface inside and covered with a transparent, perforated lid. At complete tail resorption (development stage 46), we moved the froglet into a similar new container lined with wet paper towels and a piece of egg carton as a shelter. The froglets were fed twice a week with small crickets ( *Acheta domesticus* ) sprinkled with a mixture of vitamins, minerals, and amino acids.

Phenotypic sexing was performed when the froglets were 9–12 weeks old after finishing metamorphosis (Figure 2); by this time the gonads are well differentiated and easy to observe. Taking individuals in a stratified random order which ensured that age at dissection was homogeneously distributed across populations and treatments, we humanely euthanized them in a shallow water bath of 6 g/L tricaine‐methanesulfonate (MS‐222) buffered to neutral pH with the same amount of disodium hydrogen phosphate. To ensure death, the animals were left in the bath for at least 1 h. Then we removed both feet from each individual using flame‐sterilized equipment and stored the tissue samples in 96% ethanol for genotypic sexing. We dissected the animals and recorded whether they had testes or ovaries using an APOMIC SHD200 digital microscope. Because the digestive tract often contained food remains, we cut it out, measured its mass (±0.01 g) and subtracted it from total body mass measured right before euthanasia to obtain “net body mass” (henceforth “body mass”).

Genotypic sex was diagnosed using one foot sample per individual, following the protocol described by Nemesházi et al. (2020). In short, we extracted DNA using the E.Z.N.A. Tissue DNA Kit (Omega Bio‐tek) following the manufacturer's protocol, except that digestion time was overnight. We measured DNA concentration in all samples using a NanoDrop 1000 spectrophotometer (Thermofisher Scientific). We tested all froglets for the sex marker Rds3 using high‐resolution melting (HRM; for melting curves see Figure S1 in the Supporting Information). The total HRM reaction volume was 15 μL, containing 3 μL 5× HOT FIREPol EvaGreen HRM Mix, without ROX (Solis BioDyne), 1 μL forward and 1 μL reverse primer (10 μM each), and 80–100 ng genomic DNA in MQ water to reach the final volume. Reactions were performed in a Roche LightCycler 96 qPCR Instrument, and the results were analyzed with the LightCycler 96 v.1.1.0.1320 qPCR software (Roche Diagnostics International LTD). When the Rds3 genotype did not match the phenotypic sex, the individual was tested for the sex marker Rds1 using conventional PCR, and sex reversal was accepted only if both markers confirmed sex reversal. In the present study, all individuals were successfully genotyped, and there was no mismatch between the two markers.

### 2.2 Statistical Analysis

We performed the analyses using R version 4.3.2 (R Core Team 2023). With the analysis of sex‐reversal rate, we faced several difficulties due to the nature of the data. First, our response variable was binary, and it had zero variation in certain groups; this phenomenon is called separation in binomial models. Specifically, there were no sex‐reversed individuals in 16 out of the 36 sibgroups and in the control group from one of the three woodland sites. Second, the effects of site and sibgroup are nested in the effect of habitat type. These issues make parameter estimation uncertain with the otherwise ideal model structure where habitat type is a fixed effect and site and sibgroup are nested random effects. However, simply omitting the random effects might lead to over‐estimation of the degrees of freedom. Therefore, we analysed the data in two steps.

In the first step, we explored the level of non‐independence in the data by testing various random‐effects structures to identify which groups of data are correlated. We used the ‘glmer’ function from the ‘lme4’ package to run generalized linear mixed models with binomial error. Three of the models had random intercepts only (sibgroup, or site, or sibgroup nested in site), whereas three models additionally had random slopes to allow for the effect of treatment to vary among sibgroups, or sites, or both; the seventh model had no random effects. In every model, the fixed effects were habitat type (urban or woodland), treatment (control or heated), and their interaction. The dependent variable was phenotypic sex of each individual (i.e., we modelled the likelihood of becoming male), and the dataset was restricted to genotypic females. We used likelihood ratio tests to compare model fit between nested models. When dropping a random effect significantly reduced model fit as expressed by Akaike's information criterion (AIC), we concluded that the data within the categories of that random effect were non‐independent.

In the second step, we aggregated the data by the random factor that we had identified in the first step as a source of non‐independence. Specifically, we aggregated the data by sibgroup (see Results) by calculating the proportion of sex‐reversed genotypic females for each sibgroup. We also did this separately for the control and the heat‐treated animals. We used the function ‘glm’ for running generalized linear models with binomial error to test the fixed effect of treatment separately for urban and woodland sibgroups, and we estimated the 84% confidence intervals (CI) of these effects using the ‘emmeans’ package. We compared the two CIs to test if the treatment effect differed by habitat type (note that the lack of overlap between two 84% CIs is equivalent to a significant difference, i.e., to a 95% CI around the difference that does not include zero). Then, because the treatment effects did not differ (see Results), we tested the overall effect of habitat type on the dataset aggregated only by sibgroups (i.e., sums of control and heat‐treated animals for each sex). This way, we tested three comparisons on the aggregated data; we corrected the significance values from these three tests for the false discovery rate (Pike 2011).

To compare fitness‐related traits between heat‐treated genotypic females that did versus did not undergo sex reversal, we analyzed two dependent variables: time to metamorphosis and net body mass at dissection. We did not analyze mass at metamorphosis because it correlated with the other two variables; it yielded qualitatively the same results as time to metamorphosis did (results not shown). For these analyses, we used linear mixed‐effects models, run with the ‘lme’ function of the ‘nlme’ package to allow for heteroscedasticity. We included treatment, habitat type, and sex (XX female, XX male, or XY male) as fixed factors with all their two‐way and three‐way interactions, and we allowed different variance for each combination of the fixed factors using the ‘varIdent’ function. As a random factor, we included sibgroup identity. For froglet mass, we added the age from the completion of metamorphosis as a numeric covariate (adding time to metamorphosis as an additional covariate did not change the results qualitatively; not shown). Graphical inspection of the data indicated an exponential model of growth in froglets, so we used the natural logarithm of froglet mass in the analyses, but we report the results back‐transformed to the original data scale, expressed as odds ratios. For both models, we calculated type‐2 analysis‐of‐deviance tables using the ‘Anova’ function of the ‘car’ package. Then we simplified each model by omitting all non‐significant terms except sex × treatment, and from the simplified models, we tested the difference of XX males from XX females and XY males within each treatment group using the ‘emmeans’ function. Finally, we simplified the models further by dropping all non‐significant terms to test the effects of treatment and sex without interaction.

For residual diagnostics, we used the ‘DHARMa’ package for the binomial models and standard residual plots for the linear models. These diagnostics showed that all statistical requirements were met by our data. We report mean estimates with ± standard errors (SE). The annotated script of our analyses, including R output, is available in FigShare (see Data Availability Statement).

## 3 Results

By the end of the experiment, the sample size decreased to 438 froglets. Mortality (Table 1) was similar in the heat‐treated group (36.1%) as in the control group (35%) but almost twice as high among animals that originated from urban habitats (45%) than among animals that originated from woodlands (26.1%; for further details see Figure S2 in the Supporting Information). Out of the froglets that survived to the end of the experiment, 225 (51.4%) were genotypically female, and 38 (16.9%) of those were sex‐reversed (Table 1).

**TABLE 1:** Number of individuals in each treatment group by habitat of origin.

Number of individuals	Control group		Heat‐treated group	
	Woodland	Urban	Woodland	Urban
XY male	61	45	73	34
XX female	63	47	50	27
XX male (sex‐reversed)	4	6	11	17
Died before end of experiment	49	77	45	85
Other censored observations^a^	3	5	1	17

^a^ Includes 4 froglets that escaped and 22 tadpoles that did not initiate metamorphosis.

**FIGURE 3:**
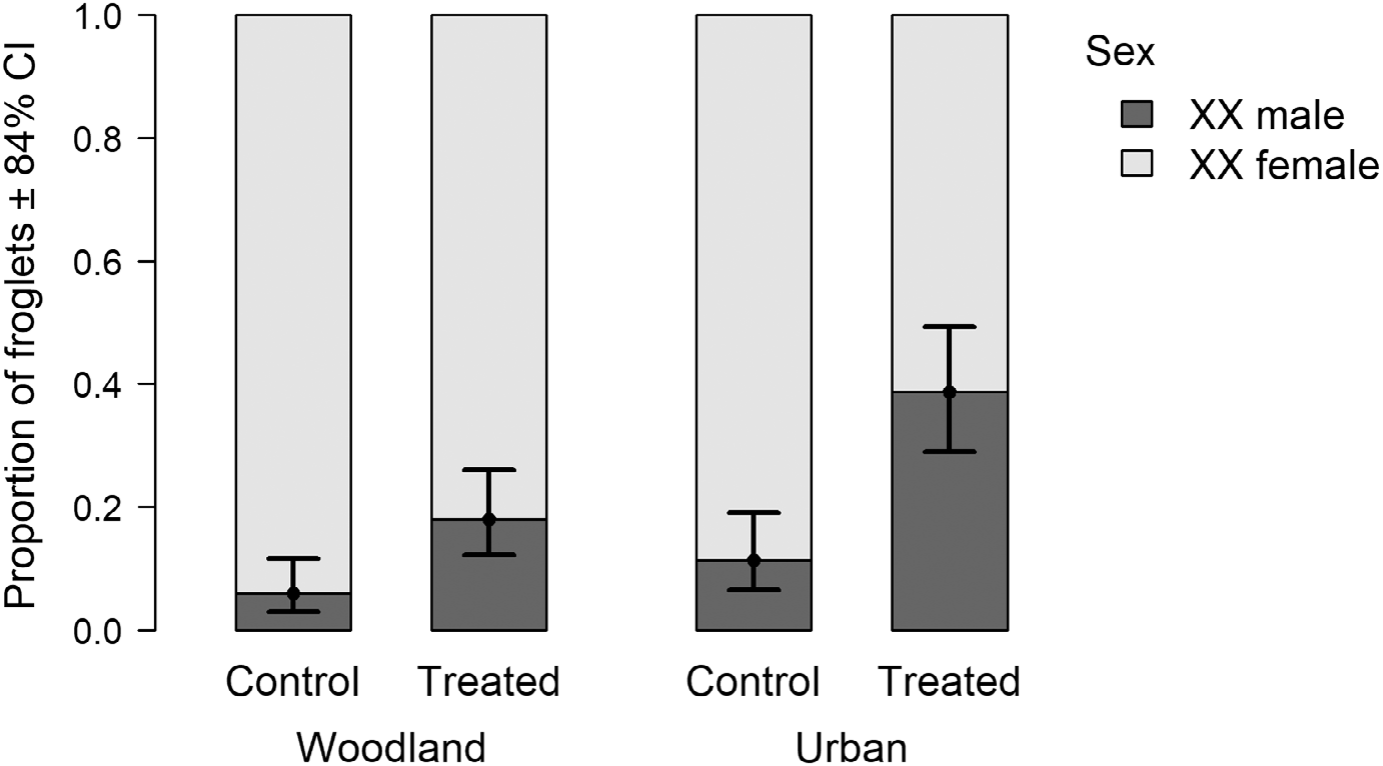
Proportion of sex‐reversed (XX male) froglets in each treatment group by habitat of origin. Barplots show the observed proportions; dots with error bars represent the model‐estimated sex‐reversal rates with 84% confidence intervals (CI).

Sex‐reversal rate differed between sibgroups, as sibgroup was significant as a random intercept although not as a random slope (Table 2). Heat treatment significantly increased sex‐reversal rate (Figure 3) in urban sibgroups (odds ratio: 4.93 ± 2.63, *p* = 0.008, 84% CI: 2.33–10.40) as well as in woodland sibgroups (odds ratio: 3.46 ± 2.13, *p* = 0.043, 84% CI: 1.46–8.21). The overlap between these two CIs indicates that the magnitude of the treatment effect (i.e., relative increment in sex‐reversal frequency in response to heat compared to the control group) did not differ significantly by habitat type. Sibgroups originating from urban habitats had significantly higher sex‐reversal rates (0.23 ± 0.04) than sibgroups originating from woodlands (0.12 ± 0.03; odds ratio: 2.34 ± 0.85, *p* = 0.029; Figure 3).

**TABLE 2:** Comparison of model fit between models with different random‐effects structures.

Model	Random effects	AIC	*p*	Likelihood ratio test for
1	(treatment|site/sibgroup)	194.7	0.894	Treatment random slope (Model 1 vs. Model 4)
2	(treatment|site)	198.0	0.992	Treatment random slope (Model 2 vs. Model 5)
3	(treatment|sibgroup)	188.7	0.577	Treatment random slope (Model 3 vs. Model 6)
4	(1|site/sibgroup)	187.8		
5	(1|site)	194.0	1	Site random intercept (Model 5 vs. Model 4)
6	(1|sibgroup)	185.8	0.004	Sibgroup random intercept (Model 6 vs. Model 4)
7	None	190.2		

**FIGURE 4:**
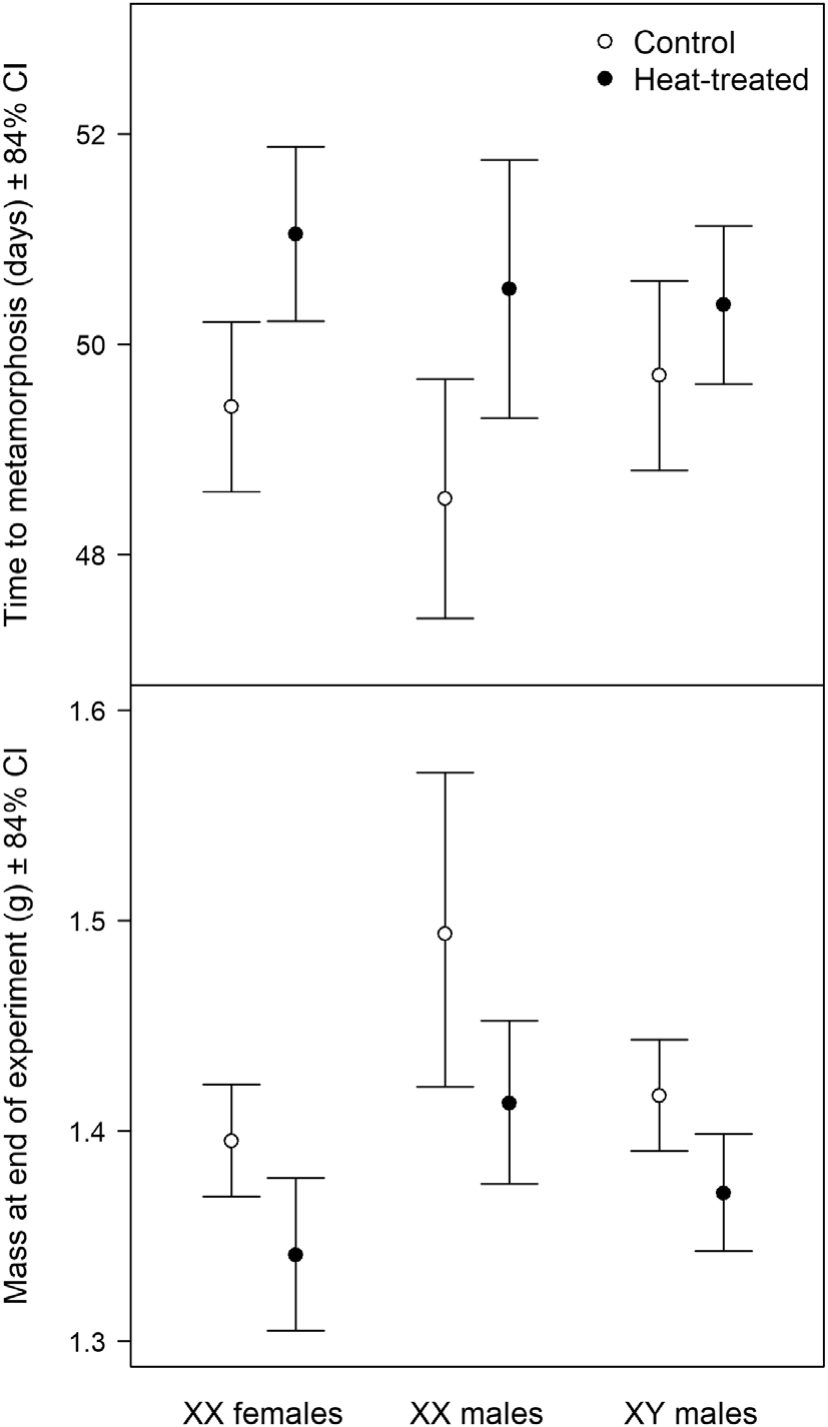
Mean values of fitness‐related traits with 84% confidence intervals (CI) by sex and treatment, estimated from linear mixed‐effects models and, for body mass, adjusted to the average age at the end of the experiment.

Heat treatment resulted in longer larval developmental time (by 1.51 ± 0.51 days, *p* = 0.003) and smaller mass at the end of the experiment (odds ratio: 0.96 ± 0.01, *p* = 0.003; Figure 4). Sex had no significant effect on the time to metamorphosis (*χ* ^2^ = 2.63, df = 2, *p* = 0.269; Figure 4), but the sexes differed significantly in mass at the end of the experiment (*χ* ^2^ = 6.82, df = 2, *p* = 0.033; Figure 4). Specifically, sex‐reversed froglets (XX males) had significantly larger mass than concordant XX females in the heat‐treated group (odds ratio: 1.05 ± 0.03, *p* = 0.044) and marginally so in the control group (odds ratio: 1.07 ± 0.04, *p* = 0.062). Combining the two treatment groups, sex‐reversed froglets had significantly larger mass than concordant females (odds ratio: 1.06 ± 0.02, *p* = 0.007). In contrast, concordant XY males had similar mass as did sex‐reversed individuals in both treatment groups (*p* > 0.146, Figure 4). These effects did not depend on the type of habitat from which the animals originated (see the annotated R script with output in FigShare as given in the Data Availability Statement).

## 4 Discussion

Our common‐garden experiment revealed that agile frogs originating from urban populations have a higher tendency for female‐to‐male sex reversal compared to their conspecifics originating from natural woodlands. This finding parallels an earlier study in which agile frogs from two urban and two woodland populations (all different from the study sites sampled in the present study) were raised in a common‐garden experiment without heat treatment, and the former showed more frequent sex reversal than the latter (Bókony, Kalina, et al. 2024). Together, these results suggest a persistent divergence in sex‐reversal propensity between habitat types, which may be due to microevolution and/or other transgenerational effects. In the present study, individuals from both habitat types showed phenotypic plasticity as evidenced by the higher sex‐reversal rate in response to larval heat exposure, but their responses were similar, such that urban individuals maintained their higher sex‐reversal rates over their woodland counterparts regardless of the thermal conditions they experienced during the sensitive period of their sex determination. This implies that urban and woodland populations have diverged in the intercept but not the slope of their thermal reaction norm of sex determination (Figure 1D). Interestingly, a similar divergence was found between highland and lowland populations of the snow skink (*Carinascincus ocellatus*), whereby both populations responded to thermal treatments, but the animals originating from the warmer lowlands always showed a higher sex‐reversal propensity than their conspecifics originating from the cooler highlands (Hill et al. 2022).

The low but non‐zero incidence of sex reversal in our control group agrees with several earlier studies on agile frogs (Bókony et al. 2021; Bókony, Kalina, et al. 2024; Mikó et al. 2021; Nemesházi et al. 2020) and suggests that some individuals have very low thresholds for sex reversal. Individual variation in thresholds is further supported by our finding here that the rate of sex reversal differed significantly among sibgroups. This variation may be due to heritability or non‐heritable parental effects (Roush and Rhen 2018). The variance components of the best‐fitting Model 6 (Table 2) yield a heritability estimate of 0.4, assuming full‐sibs, which is similar in magnitude to the heritabilities of clutch sex ratios in reptiles with TSD (Roush and Rhen 2018). If sex‐reversal propensity is indeed heritable, it can diverge between populations via microevolution, provided that sex‐reversed individuals are able to pass on their low‐threshold alleles into the next generation. The reproductive success of sex‐reversed agile frogs is not yet known, but circumstantial evidence indicates that they may be able to compete for and fertilize females, given that their adult body size is similar to concordant males, and the lack of XY offspring in some egg masses suggests a sex‐reversed sire (Nemesházi et al. 2020).

Heritable variation in sex‐reversal propensity may allow for adaptive microevolution if sex reversal influences fitness. Our results here provide support for the latter, as genotypic females that underwent sex reversal achieved slightly larger body mass by the end of our experiment compared to those that developed the female phenotype, achieving similar mass as concordant males did. Early‐life growth is an important determinant of the age and size of first reproduction and thereby affects lifetime fitness (Day and Rowe 2002). Several theoretical models explain the adaptive value of thermally sensitive sex determination by a sex‐dependent effect of temperature on the chances or speed of reaching sexual maturity (reviewed by Schwanz et al. 2016). For example, according to the “sex‐specific survival to maturity” hypothesis, the later‐maturing sex would suffer disproportionately from developing in an environment that reduces survival to maturity and, therefore, individuals developing in unfavorable environments should benefit from switching their phenotype to the earlier‐maturing sex (Schwanz et al. 2016). This hypothesis might explain female‐to‐male sex reversal in response to heat in the agile frog because females mature later than males (Sarasola‐Puente et al. 2011) and larval heat stress reduces survival (Mikó et al. 2021; Ujszegi et al. 2022). The capacity for switching phenotypic sex might be more neutral in woodland habitats where heat events are rare, but may be more advantageous in anthropogenic areas where water temperatures can get high enough to trigger sex reversal due to the urban heat island effect and lack of shading vegetation (Bókony, Balogh, et al. 2024). Thus, putting our results together, adaptive microevolution for lower sex‐reversal thresholds in anthropogenic habitats may explain the higher incidence of sex‐reversed adults in those habitats (Nemesházi et al. 2020). However, further research is needed to explicitly test the adaptive value of environmental sex reversal, not only in agile frogs but also in any species in the wild.

Alternatively, or in addition to microevolutionary changes, transgenerational plasticity could have contributed to the higher sex‐reversal propensity of urban individuals that we observed here. One likely candidate is the inheritance of epigenetic changes, which are likely a proximate mechanism of environmentally sensitive sex determination (Piferrer and Anastasiadi 2021; Whiteley et al. 2022; Zhang et al. 2022). Furthermore, parental phenotypes can also influence offspring phenotypes via the transfer of nutrients, growth regulators, or toxic contaminants into the egg (Bergeron et al. 2011; Martin and Pfennig 2010; Roush and Rhen 2018). For example, in common toads ( *Bufo bufo* ), a common‐garden experiment suggests that females living in anthropogenic habitats invest more into the protective gelatinous capsule of the eggs at the expense of reduced offspring size (Bókony et al. 2018). Similar trade‐offs might play a role in the higher sex‐reversal propensity of agile frog offspring produced by parents living in anthropogenic habitats. In this respect, it is notable that survival was lower in urban than woodland individuals in our present study, just like in a previous common‐garden experiment (Bókony, Kalina, et al. 2024). Although this might be due to maternal transfer of endocrine‐disrupting chemical pollutants (Bókony et al. 2018) or inbreeding in the populations isolated by anthropogenic landscape fragmentation (Hitchings and Beebee 1997; Lesbarrères et al. 2006), it might also emerge as a cost or side effect of “maternal programming”. For example, if urban agile frogs, similarly to toads, shift maternal resource allocation from nutrients to more protective material, the resulting handicap in offspring viability may promote sex reversal in order to “make the best out of a bad job”, assuming that shorter‐lived individuals have better chances at reproduction as males than as females because females need more time to reach the size of sexual maturity. These speculations await empirical testing.

Taken together, the results of our common‐garden experiment are in line with the hypothesis that the propensity for sex reversal is heritable and has increased by adaptive microevolution in populations living in anthropogenic habitats. This suggests that the ability to override the phenotypic sex encoded by genotypic sex determination may provide an adaptive strategy for ectothermic vertebrates to cope with the challenges of the Anthropocene. This conclusion mirrors recent empirical findings that the frequency of environmental sex reversal in reptiles varies across gradients of space and time, which may be due to local adaptations (Castelli et al. 2021; Dissanayake et al. 2021; Holleley et al. 2015). Given the huge potential for environmental sex reversal to influence the fate of populations under environmental change (Bókony et al. 2017; Nemesházi et al. 2021; Schwanz et al. 2020), we urge more research to uncover how widespread it is beyond that tiny fraction of extant species where it has been investigated so far. Such knowledge will be highly valuable for informing the conservation of ectothermic species, many of which are already threatened by extinction. For example, if heat‐induced sex reversal occurs in a population, finding out that it is advantageous means that we may not want to waste effort on protecting the animals from heat during their sex determination period. Similarly, if we find out that sex‐reversal propensity is heritable, we might help a population that has suffered a recent change in thermal conditions (e.g., due to urbanization) by introducing individuals from a population that has already evolved a relatively high capacity for sex reversal. Empirical knowledge from a wider array of species will also facilitate the parameterization of population dynamics models to forecast the fate of populations and species depending on their habitats and climatic adaptations.

## Conflicts of Interest

The authors declare no conflicts of interest.

## Benefit‐Sharing Statement

Benefits from this research accrue from the sharing of our data and results on public databases, as described above.

## Supporting information


Data S1.

## Data Availability

The data and R code supporting the results are publicly archived in the FigShare repository (DOI: 10.6084/m9.figshare.27909771).
