# Supplementary material for: Higher Sex‐Reversal Rate of Urban Frogs in a Common‐Garden Experiment Suggests Adaptive Microevolution
Source: Evol Appl. 2025 Apr 7;18(4):e70093. doi: 10.1111/eva.70093 (PMC11976026; doi:10.1111/eva.70093)

**SUPPLEMENTARY MATERIAL**

to

**"Higher sex-reversal rate of urban frogs in a common-garden experiment suggests adaptive microevolution"**

in *Evolutionary Applications*

Veronika Bókony^1*^, Emese Balogh^2^, Zsanett Mikó^1,3^, Andrea Kásler^1,3,4^, Zoltán Örkényi^2^, Nikolett Ujhegyi^1,5*^

^1^*Department of Evolutionary Ecology, Plant Protection Institute, HUN-REN Centre for Agricultural Research, Nagykovácsi út 26-30, 1029 Budapest, Hungary*

^2^*Molecular Ecology Research Group, Department of Zoology, University of Veterinary Medicine Budapest, István u. 2, 1078 Budapest, Hungary*

*^3^Department of Systematic Zoology and Ecology, ELTE Eötvös Loránd University, Pázmány Péter sétány 1/C, 1117 Budapest, Hungary*

*^4^Doctoral School of Biology, Institute of Biology, ELTE Eötvös Loránd University, Pázmány Péter sétány 1/C, 1117 Budapest, Hungary*

*^5^Department of Wildlife Biology and Management, Institute for Wildlife Management and Nature Conservation, Hungarian University of Agriculture and Life Sciences, Páter Károly u. 1, 2100 Gödöllő, Hungary*

^*^Corresponding authors:

Veronika Bókony: bokony.veronika@atk.hun-ren.hu

Nikolett Ujhegyi: ujhegyi.nikolett@atk.hun-ren.hu

**Supplementary Figure 1.** HRM-based genotyping with marker Rds3. Curves on the Normalized Melting Peaks plot were drawn by LightCycler 96 v.1.1.0.1320 (Roche Diagnostics International LTD). Besides the single nucleotide polymorphism (SNP) used for sexing, in some individuals a second SNP occurs, altering in the curves’ shape (for more details, see Fig. S2 in Nemesházi et al. 2020). →


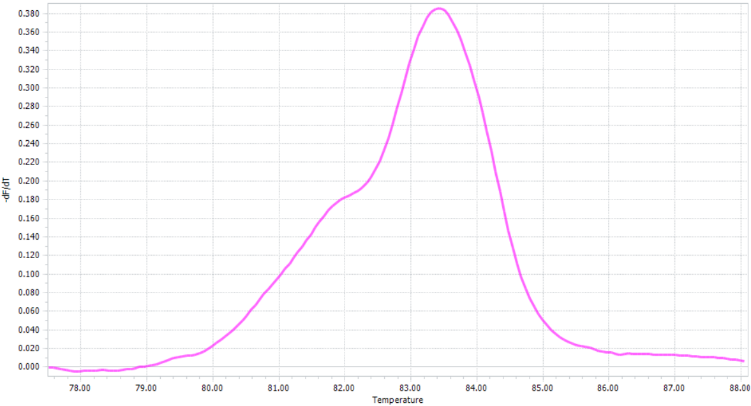

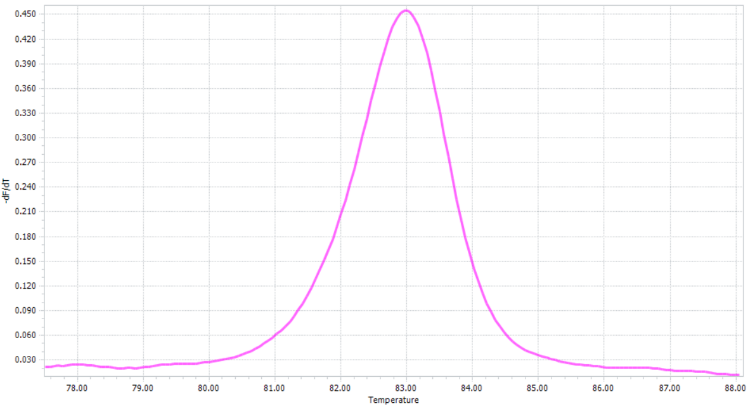

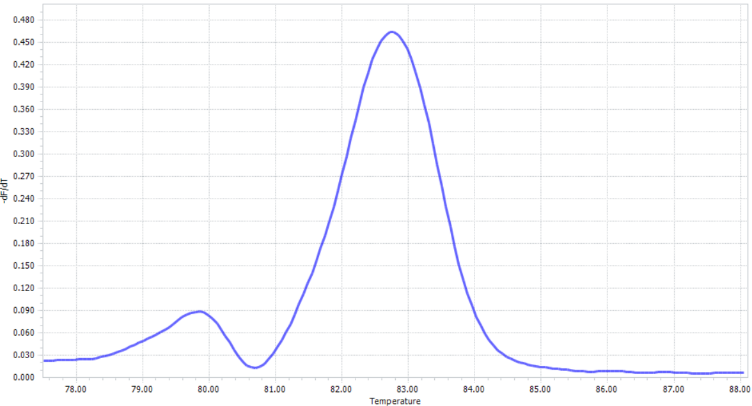

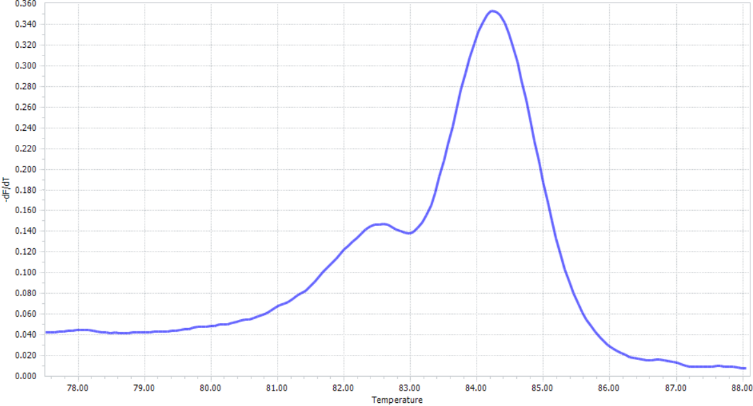


♂

1 SNP

♂

2 SNP

♀

1 SNP

♀

2 SNP

**Supplementary Figure 2.** Kaplan-Meier survival curves of survival with 95% confidence intervals by treatment and habitat of origin. Analysing these data with a mixed-effects Cox's proportional hazards model ('coxme' package), using sibgroup nested in site as random factors, the fixed effect is significant for habitat (hazards ratio: 1.99 ± 0.29, p = 0.019) but not for treatment (hazards ratio: 0.91 ± 0.21, p = 0.660) and for the interaction of habitat and treatment (hazards ratio: 1.23 ± 0.26, p = 0.430).


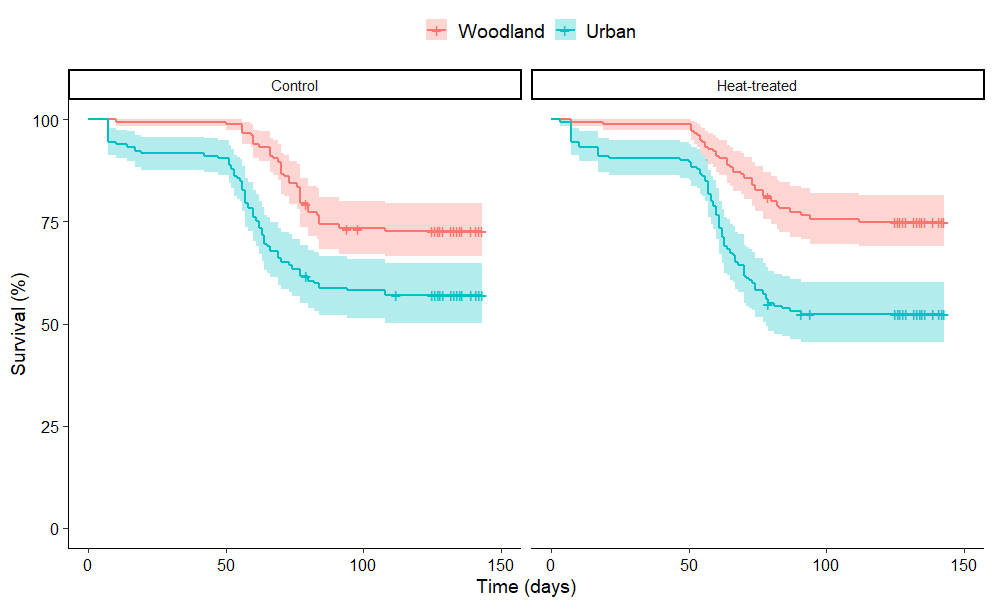

Supplement: Supplementary file 1 — Data S1. [file EVA-18-e70093-s001.docx]
